# Frequent Respiratory Viral Infections in Children with Febrile Neutropenia - A Prospective Follow-Up Study

**DOI:** 10.1371/journal.pone.0157398

**Published:** 2016-06-16

**Authors:** Martina Söderman, Samuel Rhedin, Thomas Tolfvenstam, Maria Rotzén-Östlund, Jan Albert, Kristina Broliden, Anna Lindblom

**Affiliations:** 1 Department of Medicine Solna, Infectious Diseases Unit, Center for Molecular Medicine, Karolinska Institutet and Karolinska University Hospital, Stockholm, Sweden; 2 Unit for Highly Pathogenic Viruses, Public Health Agency of Sweden; Stockholm, Sweden; 3 Department of Clinical Microbiology, Karolinska University Hospital, Stockholm, Sweden; 4 Department of Microbiology Tumor and Cell Biology, Karolinska Institutet, Stockholm, Sweden; 5 Astrid Lindgrens Children’s Hospital, Karolinska University Hospital, Stockholm, Sweden; Kliniken der Stadt Köln gGmbH, GERMANY

## Abstract

**Objective:**

Febrile neutropenia is common in children undergoing chemotherapy for the treatment of malignancies. In the majority of cases, the cause of the fever is unknown. Although respiratory viruses are commonly associated with this condition, the etiologic significance of this finding remains unclear and is therefore the subject of this study.

**Study design:**

Nasopharyngeal aspirates were collected during 87 episodes of febrile neutropenia in children age 0–18 years, being treated at a children’s oncology unit between January 2013 and June 2014. Real-time polymerase chain reaction was used to determine the presence of 16 respiratory viruses. Follow-up samples were collected from children who tested positive for one or more respiratory viruses. Rhinoviruses were genotyped by VP4/VP2 sequencing. Fisher’s exact test and Mann-Whitney U test were used for group comparisons.

**Results:**

At least one respiratory virus was detected in samples from 39 of 87 episodes of febrile neutropenia (45%), with rhinoviruses the most frequently detected. Follow-up samples were collected after a median of 28 days (range, 9–74 days) in 32 of the 39 virus-positive episodes. The respiratory viral infection had resolved in 25 episodes (78%). The same virus was detected at follow-up in one coronavirus and six rhinovirus episodes. Genotyping revealed a different rhinovirus species in two of the six rhinovirus infections.

**Conclusion:**

The frequency of respiratory viral infections in this group of patients suggests an etiologic role in febrile neutropenia. However, these findings must be confirmed in larger patient cohorts.

## Introduction

Febrile neutropenia is a common complication in children undergoing chemotherapy for the treatment of malignancies. Because septicemia (which is potentially lethal) is difficult to rule out at the onset of fever, empiric treatment with broad-spectrum antibiotics is promptly initiated based on wide indications [1]. However, in most cases, no underlying cause of the fever can be identified [2]. Febrile neutropenia is associated with long hospitalization [3–5] which has negative social effects for the child and its family [6]. In addition, hospitalization and the use of broad-spectrum antibiotics increase the patient’s risk of subsequent infection with antibiotic-resistant bacteria [7,8] and fungal infections [9,10]. A better understanding of the etiology of febrile neutropenia is thus needed in order to decrease unnecessary hospitalization and excessive antibiotic use.

As infections with respiratory viruses are a common morbidity in children [11] respiratory viruses likely play a significant role in childhood febrile neutropenia. Advances in molecular methods have increased the sensitivity of viral diagnostics tests, with recent studies reporting the detection of respiratory viruses in the nasopharynx in 44–57% of childhood febrile neutropenia episodes using real-time polymerase chain reaction (PCR) [3–5,12]. However, the clinical significance of positive PCR findings is unclear, as respiratory viruses have also been detected in asymptomatic immunocompetent children [13–16]. In addition, some respiratory viruses can be detected weeks after the first infection, which is suggestive of prolonged viral shedding [17,18]. Here, we describe the results of a longitudinal study involving repeated sampling and the assessment of a broad panel of respiratory viruses by PCR to clarify whether respiratory viruses play a causal role in childhood febrile neutropenia.

## Methods

### Study group

Children aged 0–18 years, who were treated for a malignancy at the childhood cancer unit at Astrid Lindgren Children’s Hospital in Stockholm, Sweden, between January 2013 and June 2014, were eligible for enrollment in this study. All the patients who met the criteria for febrile neutropenia were asked to participate. Patients could be enrolled multiple times if recurrent episodes of febrile neutropenia occurred during the study period. To be enrolled for a new episode, the patient needed to have been afebrile for more than 72 hours and have completed the antibiotic treatment for the previous episode of febrile neutropenia. Febrile neutropenia was defined as a body temperature of ≥38.5°C on one occasion or ≥38.0°C on two occasions at least 60 minutes apart, combined with an absolute neutrophil count of either ≤0.5×10^9^/L on one occasion or ≤1.0×10^9^/L with a decline to less than ≤0.5×10^9^/L over a subsequent 48-hour period. Oral and written information regarding the study were provided to each patient prior to enrollment, and signed consents were obtained from their caretakers. The study was approved by the Regional Ethical Review Board in Stockholm.

### Sample collection and microbiological analyses

A nasopharyngeal aspirate (NPA) was collected within 72 hours of the onset of fever. The NPA was diluted in approximately 2 mL of saline and immediately sent to the accredited Karolinska University Laboratory (ISO: 15189:2012) for microbiological analysis. Viral nucleic acids were extracted from the NPA with a MagAttract Virus Mini M48 kit (Qiagen, Sollentuna, Sweden) and analyzed with in-house real-time PCRs for the following 16 viruses: adenovirus (HAdV); bocavirus (HBoV); coronaviruses NL63/OC43/229E/HKU1 (HCoV); enterovirus (EV); influenza virus A, including A(H1N1)pdm09 and B (Flu); metapneumovirus (HMPV); parainfluenza viruses 1–3 (PIV); respiratory syncytial virus (RSV) and rhinovirus (RV) [19]. A semi-quantitative assay was used and the actual Cycle thresholds-values (Ct-values) were provided, however, the NPA has not been validated for quantitative data. In all study subjects who initially tested positive for one or more respiratory viruses, a follow-up sample was collected at the time of their next visit to the hospital. This follow-up sample was collected regardless of the patient’s absolute neutrophil count or symptoms and was analyzed in the same manner as the first sample. However, if the patient had a new episode of febrile neutropenia at the time of the follow-up, this sample was considered both a follow-up sample and a new episode sample, which required an additional follow-up sample if the new episode tested positive for a respiratory virus.

RV/EV genotyping was performed by VP4/VP2 sequencing for samples that were PCR-positive for RV and/or EV using a recently published method [20] based on a method and primers originally published by Wisdom *et al*. [21]. Briefly, viral RNA was extracted as described above for most samples, but the RNeasy Lipid Tissue Mini Kit (Qiagen, Sollentuna, Sweden) was used for samples that were negative in the VP4/VP2 PCR after MagAttract extraction. The RNA was used for the nested reverse transcriptase (RT)-PCR with One-step Superscript-Platinum Taq (Life Technologies, Stockholm, Sweden) for the first (outer) PCR, and Platinum Taq for the second (nested) PCR. Sequencing was done on an ABI 3730 instrument and the RV/EV species and type was determined by maximum likelihood phylogenetic trees constructed using PhyML [22] and the sequences have been deposited in GenBank under accession numbers KX15472-KX154585 for RV-A and KX290514-KX290520 for RV-C. Two of the RV-C samples were not submitted to the GenBank because the sequences were of suboptimal quality due to a probable infection with more than one RV genotype.

Blood cultures were collected from all patients for the detection of bacterial infections and analyzed at the Karolinska University Laboratory, as per routine clinical procedures.

### Clinical data collection

Clinical data, including the results of biochemical and microbiological analyses, patient’s characteristics, treatment during the febrile episode, fever characteristics, and the duration of hospitalization, were collected from the medical records.

### Statistical analyses

Data were analyzed using GraphPad Prism 6.0 software (GraphPad Prism, San Diego, CA). Fisher’s exact test and the Mann-Whitney U test were used for group comparisons of categorical and continuous data, respectively. A p-value less than 0.05 was considered statistically significant.

## Results

### Study population

A total of 56 patients representing 92 episodes of febrile neutropenia were enrolled in the study. Five episodes were excluded due to incomplete sampling. Therefore, the analyses included 54 patients with 87 episodes of febrile neutropenia (ranges, 1–5 episodes per patient and 8–214 days between episodes). The characteristics of the study participants are listed in Table 1. The median age across all episodes was 7 years (range, 0.5–17.7 years), and 59% of the episodes occurred in females. The patient was undergoing treatment for a hematologic malignancy in 51% of the episodes and for a solid tumor in 49% of the episodes. Respiratory symptoms were noted in 70% of the episodes of febrile neutropenia (Table 1).

**Table 1:** Characteristics of 87 episodes of febrile neutropenia.

	Episodes of febrile neutropenia	Single respiratory viral infection	Multiple respiratory viral infection	Co-presence of respiratory virus and septicemia	Only septicemia	Fever of unknown origin	P-value Only Respiratory viral infection^a^ vs. only septicemia	P-value Only Respiratory viral infection^a^ vs. fever of unknown origin
**Episodes**	n = 87	n = 34 (39%)^b^	n = 2 (2%)	n = 3 (3%)	n = 5 (6%)	n = 43 (49%)^c^		
**Age (years)**	7.0 (0.5–17.7)	7.9 (0.8–16.9)	2.4 (1.6–3.2)	4.6 (0.8–4.9)	6.0 (1.5–12.1)	7.6 (0.5–17.7)	0.78	0.73
**Gender**							1	0.18
**Male**	36 (41%)	11 (32%)	1 (50%)	1 (33%)	2 (40%)	21 (49%)		
**Female**	51 (59%)	23 (68%)	1 (50%)	2 (67%)	3 (60%)	22 (51%)		
**Tumor type**							0.14	0.04
**Solid**	43 (49%)	14 (41%)	1 (50%)	0 (0%)	0 (0%)	28 (65%)		0.83
**Hematologic**^d^ **malignancy**	44 (51%)	20 (59%)	1 (50%)	3 (100%)	5 (100%)	15 (35%)		
**Days of hospitalization**	5 (0->30)	5 (0–13)	7 (4–9)	7 (6–10)	11 (8–22)	5 (2->30)	<0.0001	
**Days with antibiotics**	7 (0–30)	7 (0–13)	7 (4–9)	13 (9–17)	15 (14–17)	7 (0–30)	<0.0001	0.93
**Days with fever**	2 (1–16)	2 (1–6)	4 (2–6)	3 (1–5)	4 (2–10)	2 (1–16)	0.08	0.91
**Temperature**^e^	39.1 (38.0–40.5)	39.1 (38.0–40.5)	39.3 (38.8–39.8)	39.0 (38.7–39.2)	39.7 (38.7–40.3)	39.0 (38.1–40.2)	0.08	0.86
**CRP (mg/L)**^e^	83 (4–412)	80 (4–332)	134 (15–253)	114 (80–163)	207 (41–341)	81 (4–412)	0.05	0.6
**Respiratory symptoms**	n = 61 (70%)	n = 29 (85%)	n = 2 (100%)	n = 3 (100%)	n = 2 (40%)	n = 25 (58%)	0.04	0.007

The figures represent median value and range for age, days of hospitalization, days with antibiotics, days of fever and temperature

^**a**^ single respiratory viral infection group combined with multiple respiratory viral infection group

One patient was also positive for symptomatic *Clostridium difficile*

^c^ Two patients from this group had local infections with clinical significance, one with *Pseudomonas aerguinosa* from a local wound culture and one with *Staphylococcus aureus* and *streptococcus group G* in local culture from a gastrostomy

^d^ Hematological malignancies include acute lymphatic leukemia, acute myeloid leukemia, Hodgkins lymphoma and non-Hodgkins lymphoma

^e^ Maximum value during the episode of febrile neutropenia

Abbreviations: CRP, C-reactive protein

### Presence of respiratory viruses

At least one respiratory virus was identified in 39 (45%) of the 87 episodes. A single respiratory virus was detected in 34 (39%) of the episodes (Table 1). Multiple viruses (range, 2–4 viruses) were detected in two episodes (2%), whereas co-presence with a respiratory virus and septicemia was detected in three episodes (3%) (Table 1). Of the 87 episodes, RV was the most frequently detected respiratory virus (n = 21, 24%), followed by HCoV (n = 7, 8%), Flu (n = 4, 5%), RSV (n = 3, 3%), PIV (n = 3, 3%), HMPV (n = 2, 2%), HBoV (n = 2, 2%), and HAdV (n = 1, 1%) (Table 2). In 12 of the virus-positive episodes, the patient was <4 years of age. The most common respiratory virus identified in the group <4 years of age was RV (n = 9), followed by PIV (n = 2), HCoV (n = 2), HAdV (n = 1), HBoV (n = 1) and RSV (n = 1). In addition, one episode with co-presence with a respiratory virus and septicemia was identified in the group <4 years of age. Of the 36 episodes involving only respiratory viral infection (i.e., infection with either a single respiratory virus or multiple respiratory viruses) respiratory symptoms were detected in 31 (86%) (Table 1). No symptoms were apparent in four episodes involving RV and one episode involving HCoV (Table 2). Of the 31 episodes involving respiratory symptoms, 26 reported the appearance of respiratory symptoms at time of fever onset (Table 2). In the remaining five episodes, the respiratory symptoms appeared over 6 days before the onset of fever. These five episodes represented one Flu B and four RV (Table 2).

**Table 2:** Findings of respiratory viruses in 39 of the 87 episodes of febrile neutropenia.

	Total respiratory viruses (n = 43)	Single respiratory viral infections (n = 34)	Multiple respiratory viral infections (n = 6^a^)	Co-presence of respiratory virus and septicemia (n = 3)	Respiratory symptoms (new symptoms correlated to the detection of the virus and the fever debut yes/no)	CT-level PCR	Respiratory viral clearance at follow-up (32 of 39 re-sampled)	Median time to follow-up (days, range)
HAdV	1 (1%)	0	1	0	Runny nose, cough (y)	32.00	1/1	28
HBoV	2 (2%)	0	1	1	Runny nose, cough (y)	26.67	2/2	40 (28–51)
HBoV					Runny nose (y)	35.34		
HCoV NL63	3	2	1	0	Runny nose, coughing (y)	25.78	3/3	16 (13–20)
					Runny nose, coughing, hoarse (y)	33.13		
					Runny nose, coughing (y)	32.7		
HCoV 229E	1	1	0	0	Runny nose, coughing, sore throat, muscle and joint pain (y)	19.09	1/1	10
HCoV HKU1	3	3	0	0	Coughing (n), tachypnea(y)	21.18	2/3	24 (15–60)
					No symptoms	18.92		
					Runny nose, coughing (y)	20.55		
HMPV	2 (2%)	2	0	0	Runny nose (n), coughing (y)	33.86	2/2	25 (18–32)
					Runny nose, coughing (y)	28.70		
Flu A	2	2	0	0	Coughing, needing oxygen, headache, joint and muscle pain (y)	22.84	2/2	25 (19–31)
					Runny nose, coughing (y)	29.37		
Flu B	2	2	0	0	Coughing, sore throat, hoarse, headache (y)	35.17	1/1	41
					Runny nose, sore throat, coughing (n)	24.94		
PIV 2	1	1	0	0	Runny nose, coughing (y)	27.72	1/1	31
PIV 3	2	2	0	0	Sore throat (y), coughing (n)	35.77	1/1	23
					Runny nose, coughing (y)	18.44		
RSV	3	2	1	0	Runny nose, coughing (y)	24.87	2/2	33 (28–38)
					Runny nose (n), coughing (y), hoarse (y)	37.92		
					Runny nose, coughing (y)	17.96		
RV A	11	7	2	2	Runny nose, coughing (n)	25.96	5/9	30 (12–63)
					Runny nose, coughing (n)	21.59		
					Runny nose (n)	32.76		
					Runny nose, coughing (y)	27,36		
					Coughing (y)	25.59		
					No symptoms	33.78		
					Runny nose, coughing (y)	24.36		
					Coughing (y)	26.11		
					Runny nose, coughing (y)	27.42		
					Runny nose, coughing (y)	24.17		
					Runny nose, coughing (y)	26.58		
RV C	9	9	0	0	Runny nose, sore throat, hoarse, vesicles on the tongue, headache (y)	37.17	7/7	28 (9–74)
					Runny nose, coughing (n)	21.43		
					Coughing (y)	24.39		
					Runny nose (y)	22.62		
					No symptoms	27.84		
					Runny nose (y)	23.78		
					Runny nose (y)	23.82		
					No symptoms	32.94		
					Runny nose (y)	30.36		
Unknown species	1	1	0	0	No symptoms	33.14	1/1	10

^**a**^ One episode with RV and HCoV NL63 and one episode with HAdV, HBoV, RSV and RV.

Abbreviations: Flu, Influenza virus; HAdV, Human adenovirus; HBoV, Human bocavirus; HCoV, Human coronavirus; EV, Enterovirus; HMPV, Human metapneumovirus; RV, Rhinovirus; RSV, Respiratory syncytial virus; PIV, Parainfluenza virus

### Presence of septicemia

Bacterial blood cultures were positive in 13 (15%) of the episodes of febrile neutropenia. Five cultures were excluded from further analysis and not defined as septicemia because they were determined to be either contaminants or of no clinical relevance by the treating clinician and/or the laboratory: *Micrococcus species* (n = 1), *coagulase-negative staphylococcus* (n = 1), *Staphylococcus epidermidis* (n = 2), and unspecified gram-positive bacteria (n = 1). Eight episodes were therefore considered true septicemia. Of these, five episodes involved only septicemia (6%) (Table 3) (i.e. they tested negative for respiratory viruses by PCR). Three episodes involved co-presence with a respiratory virus and septicemia; gram-positive bacteria (*Staphylococcus epidermidis* (n = 2) and *alpha streptococcus* (n = 1)) were detected in all three episodes and one episode was also positive for gram-negative bacteria (*Escherichia coli*). In all three episodes involving co-presence of respiratory virus and septicemia the patients were being treated for a hematologic malignancy, and all had respiratory symptoms (Table 1). The viruses found in this group were RV (n = 2) and HBoV (n = 1) (Table 2).

**Table 3:** Septicemia results.

	Septicemia 1	Septicemia 2	Septicemia 3	Septicemia 4	Septicemia 5
Age (years)	1.5	12.1	4.5	6.0	9.4
Gender	Male	Male	Female	Female	Female
Type of bacteria	Staphylococcus epidermidis	Coagulase negative staphylococcus	Escherichia Coli	Staphylococcus Aureus	Staphylococcus Aureus
Tumor type	Hematologic	Hematologic	Hematologic	Hematologic	Hematologic
Days of hospitalization	11	14	8	22	11
Days with antibiotics	17	15	14	15	16
Days of fever	4	10	2	10	2
Temperature ^a^	39.4	39.7	40.3	39.8	38.7
CRP (mg/L) ^a^	141	341	207	321	41
Respiratory symptoms	Yes	Yes	No	No	No

^a^ Maximum value during the episode of febrile neutropenia

Abbreviations: CRP, C-reactive protein

### Fever of unknown origin

In 43 episodes (49%), no respiratory virus or septicemia were detected and the febrile episode was therefore defined as a fever of unknown origin (Table 1).

### Group comparisons

When comparing episodes involving only respiratory viral infection (i.e., infection with either a single respiratory virus or multiple respiratory viruses) with episodes involving only septicemia or fever of unknown origin, no statistically significant differences were observed with respect to age, gender, days with fever, or maximum temperature (Table 1). However, the presence of respiratory symptoms was significantly higher in the episodes involving only respiratory viral infection (86%) compared with both the episodes involving only septicemia (40%) and fever of unknown origin (58%) (p = 0.043 and p = 0.007, respectively). The episodes with only respiratory viral infection were also more often treated for a hematological malignancy (58%) compared to the fever of unknown origin episodes (35%) (p = 0.04). The episodes involving only septicemia had significantly more days on antibiotics (median, 15 days; range, 14–17 days) (p<0.0001) and a longer hospitalization (median, 11 days; range, 8–22 days) (p<0.0001) compared with the episodes with only respiratory viral infection (median, 7 days; range, 0–13 days versus median, 5 days; range, 0–13 days, respectively). The episodes involving only septicemia had a higher maximum CRP-median (median, 207 mg/L; range, 41–341 mg/L) compared with the episodes involving only respiratory viral infection (median, 80 mg/L; range, 4-332mg/L), but the difference was not statistically significant (p = 0.05).

### Virus clearance at follow-up

Follow-up samples were collected for 32 of the 39 virus-positive episodes. Eight of these samples represented a new episode of febrile neutropenia. The infection had resolved in 25 of the 32 episodes (78%) (median, 28 days; range, 9–74 days). At follow-up, six episodes exhibited persistent RV and one episode exhibited persistent HCoV with a median follow-up time of 31 days (range, 12–74 days) and 24 days, respectively. Of these seven episodes, three involving RV presented with a new episode of febrile neutropenia, and all except the episode involving HCoV presented with new or persistent respiratory symptoms (Table 4). A new respiratory virus was detected in six follow-up samples: Flu A (n = 2), RV (n = 2), HCoV (n = 1), and RSV (n = 1). Of these six, the episodes for all except one involving RV exhibited respiratory symptoms, and one (Flu) represented a new episode of febrile neutropenia.

**Table 4:** Viruses detected at time of follow up.

Rhino/corona virus species detected at time of febrile neutropenia	CT value PCR	Respiratory symptoms (new symptoms correlated to the detection of the virus and the fever debut yes/no)	Rhino/corona virus species detected at time of follow up (days after first sampling)	CT value PCR	Respiratory symptoms (new symptoms correlated to the detection of the virus yes/no)
A61	21.59	Runny nose, coughing (n)	A61 (12)	32.76	Runny nose, coughing (n)
A61	32.76	Runny nose, coughing (n)	C16 (30)	22.62	Runny nose (n)
C16	22.62	Runny nose (n)	A101 (74)	26.73	Runny nose (y)
A65	25.59	Runny nose, coughing (y)	A65 (51)	30.99	Runny nose (y)
A67	33.78	No symptoms	A67 (14)	26.11	Cough (y)
A39	27.42	Runny nose, coughing (y)	A39 (32)	28.94	Cough (y)
HKU1	21.18	Coughing, tachypnea (y)	HKU1 (24)	21.35	No symptoms

Abbreviations: y, yes; n, no.

### Genotyping of RV–PCR positive samples

**Fig 1:**
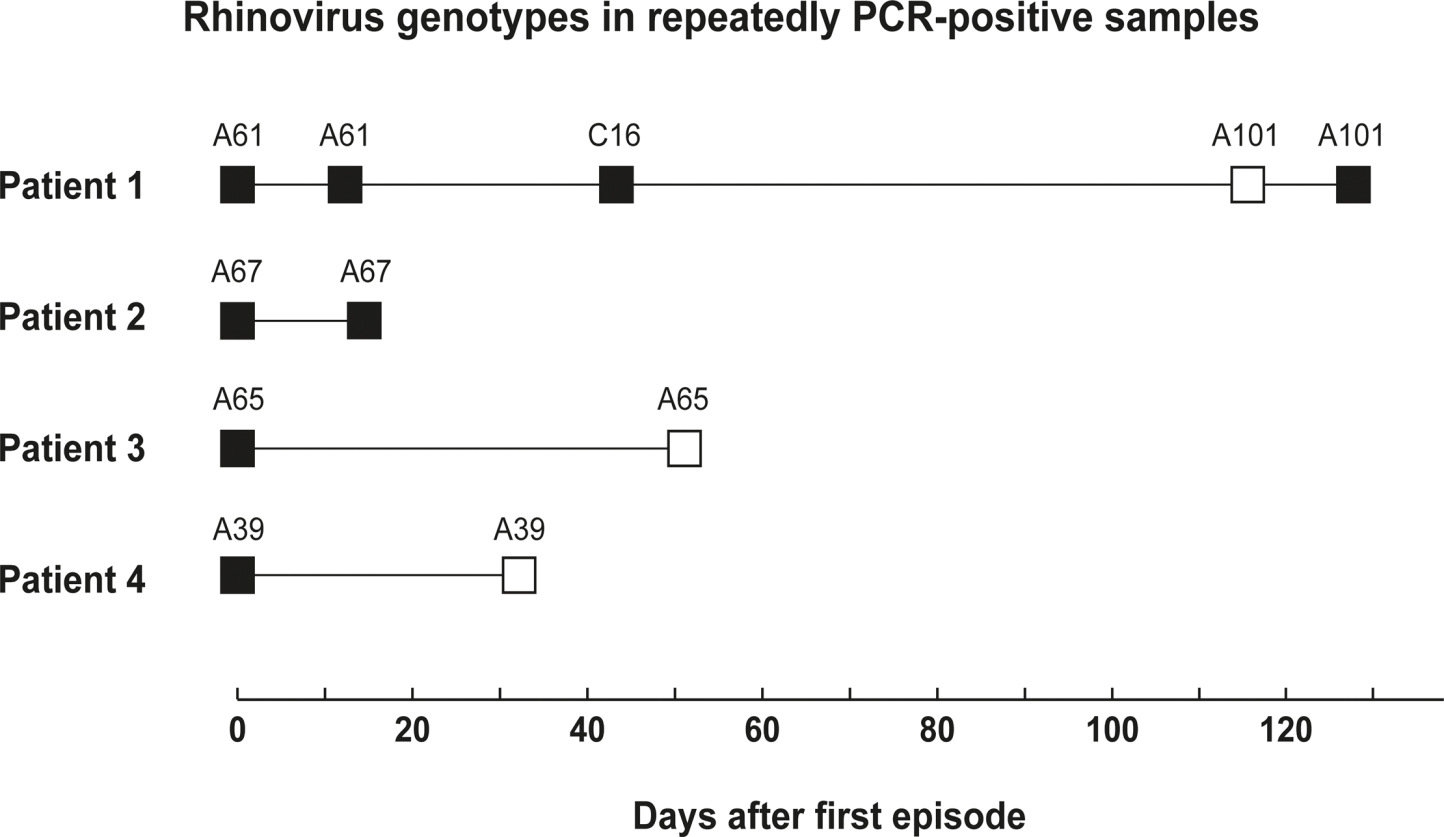
Genotyping of repeatedly rhinovirus positive samples collected during episodes of febrile neutropenia (black boxes) and at follow-up (white boxes).

RV genotyping was performed to determine whether the episodes exhibiting repeated PCR positivity involved a new or persistent infection. Of samples from the 21 RV-positive episodes, 20 were successfully sequenced, resulting in the identification of 11 RV-A and 9 RV-C species (Table 2). Before sequencing, four patients, representing six episodes, remained RV-positive at follow-up (Table 4). One patient was RV-positive in three follow-up samples; however sequencing revealed that two of these follow-up samples contained a new RV specie (Fig 1). Sequencing also revealed that the three remaining patients all had the same genotype (Fig 1). All of the RV-C had cleared by the time of follow-up (Table 2), and the four persistent episodes involving RV were associated with RV-A infections (Table 2).

## Discussion

To the best of our knowledge, this is the first longitudinal study to assess respiratory viral persistence in children presenting with febrile neutropenia. We found that respiratory viral infections are common in children with febrile neutropenia, in the majority of episodes accompanied by respiratory tract symptoms and that the infection had resolved at follow-up in the majority of the episodes. These results support the theory holding that there is a causal relationship between respiratory viral infections and episodes of febrile neutropenia, but proving this theory will require more longitudinal studies with asymptomatic neutropenic control cohorts. In addition, prolonged viral persistence with regard to these respiratory tract infections seems to be uncommon in children undergoing chemotherapy for the treatment for malignancies.

In a previous study we detected respiratory viruses in 46% of episodes of febrile neutropenia [3]. Subsequent studies have confirmed this finding, with detection rates ranging between 52–57% [4,5]. The detection rate of 45% in our current study is consistent with earlier studies.

It is crucial to correctly diagnose febrile neutropenia in immunosuppressed children, as infections with viruses [23,24], bacteria [25,26] or fungi can be fatal [26] in this group of patients. The clinical significance of a single time-point PCR finding of certain pathogens has been debated. Studies on immunocompetent children have reported that viruses such as HBoV, HCoV, and RV are detected in asymptomatic children at rates ranging between 27–40% [13–16], which could be suggestive of prolonged viral shedding from an earlier infection or the incubation period prior to a symptomatic- [17], or asymptomatic infection [13,14,17]. To clarify this issue, more longitudinal studies with appropriate control groups are needed. Rhedin *et al*. conducted a case-control study to investigate the etiologic role of respiratory viruses in a group of children with respiratory tract symptoms seeking medical care at a pediatric emergency center [13]. The control group consisted of children on routine visits to child welfare centers for vaccination within the childhood immunization program. The detection rate of respiratory viruses in the control group was high (35.4%) with RV and HCoV the most commonly detected viruses in asymptomatic children and HMPV, PIV and RSV detected only rarely. The authors concluded that HMPV, PIV and RSV play an etiologic role and suggested that findings of RV and HCoV must be carefully interpreted [13].We chose a longitudinal design for the present study, and found that 78% of the infections had resolved by the median follow-up time of 28 days. In addition, two of the RV infections identified at follow-up represented new species of RV from the previous infection, which increases the proportion of resolved infections to 84%.

One major limitation of the current study was the lack of a control cohort of neutropenic patients without fever to address the question as to whether respiratory viruses are frequently found in asymptomatic immunosuppressed children. However, the use of a longitudinal design allowed us to collect follow-up samples from patients both with and without respiratory symptoms. Only five patients at the first sampling and two patients at follow-up sampling were positive for a respiratory virus without the presence of respiratory symptoms, which suggests that the frequency of asymptomatic infections is low in this group of patients. Interestingly, RV was detected in five of these seven episodes and HCoV was detected in the other two, which are in line with other studies [13]. The presence of respiratory symptoms was significantly higher in the episodes involving only respiratory viral infection, compared with both episodes involving only septicemia and fever of unknown origin, with symptoms appearing with the onset of fever in the majority of the episodes. Peck *et al*. investigated the incidence of respiratory virus in patients receiving a hematopoietic stem cell transplant using longitudinally collected respiratory tract samples regardless of symptoms [27]. In line with our results, a majority of the respiratory viruses detected could be correlated with respiratory tract symptoms, with the exception of PIV, which was detected in asymptomatic patients. Infection with RV or HCoV was not investigated in that study. In addition, high viral loads were correlated to more symptoms. Our real-time-PCR technique was not validated for use in reporting clinical viral loads; thus the presented Ct-values must be interpreted with extreme caution. Nevertheless, the Ct-values <30 observed in 27 of the 39 episodes of febrile neutropenia are suggestive of high viral loads (Table 2). Considered together, our data suggests that respiratory viruses play an etiologic role in febrile neutropenia, but our results should be interpreted carefully, especially those regarding RV, which is commonly detected in asymptomatic patients[13].

Despite the above-mentioned limitations, we believe that our findings of a respiratory virus together with other clinical parameters such as respiratory symptoms, negative blood cultures after 48 hours, and a history of short duration of myelosuppression, may help to reduce the treatment time with broad-spectrum antibiotics and the need for prolonged hospitalizations.

We were particularly interested in RV in our study because it was the most frequently detected virus, was still detected at follow-up, and has been reported in asymptomatic immunocompetent children [13–16]. The clinical impact of RV has been correlated with severe respiratory diseases in children under 5 years of age [28]. In our study, only the A and C species of RV were identified. These species are believed to be more pathogenic and more strongly associated with hospital treatment than RV-B [28–30]. RV-A and RV-C causing both upper and lower respiratory tract infections were the most frequently detected species in another study that investigated RV infection in immunosuppressed children [31]. In the only case of RV-B reported in that study, the patient had an upper respiratory tract infection. In our study cohort, all episodes with a detected RV infection were hospitalized as a result of their febrile neutropenia, and therefore the severity of the disease in relation to the RV specie was difficult to evaluate.

Respiratory viruses and their shedding times have not been thoroughly investigated in immunosuppressed children. Earlier studies on RSV have shown prolonged viral shedding in immunosuppressed children compared with healthy children [32]. Martin *et al*. conducted a longitudinal study of healthy immunocompetent children attending a daycare center [33] and found prolonged shedding (i.e., persistent virus >7 days) of all viruses examined except Flu A and B. Jartti *et al*. reported shedding times of 2–3 and 5–6 weeks for EV and RV, respectively, in immunocompetent children [17]. However, RV was not sequenced in that study; therefore, it is unclear whether the sample at 5 weeks represented the same or a new genotype of RV. Another study reported that prolonged persistence (>30 days) of the same RV strain is uncommon (<5%) [34]. Shedding times were of special interest in our cohort of immunosuppressed children because viral shedding necessitates isolation from other immunosuppressed children. The design of our present study did not allow us to determine the exact shedding time, which is a major limitation. However, the clearance of all viruses except RV-A and HCoV at a median follow-up time of 28 days, together with respiratory symptoms appearing at the time of fever onset in a majority of the patients, suggests that the shedding time for viruses such as Flu, HMPV, RSV and PIV is limited. The same RV genotypes were detected from follow-up samples in four episodes, with a follow-up time of 12–51 days. Furthermore, five patients positive for RV reported symptom appearance 6 days or longer before fever onset. That could indicate longer shedding times for RV, which is in line with results from studies on immunocompetent children [17]. However, this needs to be addressed in additional studies with repeated follow-up sampling, preferable on a weekly basis.

HBoV has been associated with acute wheezing in immunocompetent children [35]. In immunosuppressed children, HBoV has been detected both together with other viruses and also detected repeatedly, suggesting prolonged shedding or reactivation [36,37]. In this study, HBoV was detected twice, with HAdV, RSV and RV in one episode and together with septicemia in another episode; both episodes involved respiratory symptoms. At follow-up sampling, HBoV was no longer detectable. In our previous study [3], we detected HBoV in three episodes: together with one other virus (RV) with septicemia and as the only agent, making it difficult to ascertain the clinical relevance.

## Conclusion

Our data strengthen the evidence suggesting that respiratory viruses play an etiologic role in febrile neutropenia in children receiving treatment for a malignancy. If confirmed in future studies, the findings reported here will have implications for the clinical management of these patients. Our finding could lead to a decrease in the duration of both hospitalization and treatment with broad-spectrum antibiotics, leading to positive social effects for the child and his or her family as well as decreasing the risk of subsequent infection with antibiotic-resistant bacteria.
